# Genetic, Maternal and Placental Factors in the Association between Birth Weight and Physical Fitness: A Longitudinal Twin Study

**DOI:** 10.1371/journal.pone.0076423

**Published:** 2013-10-23

**Authors:** Robbert N. H. Touwslager, Marij Gielen, Frans E. S. Tan, Antonius L. M. Mulder, Willem J. M. Gerver, Luc J. Zimmermann, Alfons J. H. M. Houben, Maurice P. Zeegers, Catherine Derom, Robert Vlietinck, Hermine H. Maes, Coen D. A. Stehouwer, Martine Thomis

**Affiliations:** 1 Department of Pediatrics, Maastricht University Medical Centre, Maastricht, The Netherlands; 2 School for Oncology and Developmental Biology, Maastricht, The Netherlands; 3 Nutrition and Toxicology Research Institute Maastricht, Maastricht, The Netherlands; 4 Section of Complex Genetics, Department of Genetics and Cell Biology, Maastricht University, Maastricht, The Netherlands; 5 Department of Methodology and Statistics, Maastricht University, Maastricht, The Netherlands; 6 Care and Public Health Research Institute, Maastricht, The Netherlands; 7 Department of Internal Medicine, Maastricht University Medical Centre, Maastricht, The Netherlands; 8 Cardiovascular Research Institute Maastricht, Maastricht, The Netherlands; 9 Department for Human Genetics, Faculty of Medicine, Catholic University of Leuven, Leuven, Belgium; 10 Department of Kinesiology, Faculty of Kinesiology and Rehabilitation Sciences, University of Leuven, Leuven, Belgium; 11 Department of Human and Molecular Genetics, Virginia Institute of Psychiatric and Behavioral Genetics, Virginia Commonwealth University, Richmond, Virginia, United States of America; University of Hong Kong, Hong Kong

## Abstract

**Background:**

Adult cardiorespiratory fitness and muscle strength are related to all-cause and cardiovascular mortality. Both are possibly related to birth weight, but it is unclear what the importance is of genetic, maternal and placental factors in these associations.

**Design:**

Peak oxygen uptake and measures of strength, flexibility and balance were obtained yearly during adolescence (10–18 years) in 114 twin pairs in the Leuven Longitudinal Twin Study. Their birth weights had been collected prospectively within the East Flanders Prospective Twin Survey.

**Results:**

We identified linear associations between birth weight and adolescent vertical jump (b = 1.96 cm per kg birth weight, *P* = 0.02), arm pull (b = 1.85 kg per kg birth weight *P* = 0.03) and flamingo balance (b = −1.82 attempts to stand one minute per kg birth weight, *P* = 0.03). Maximum oxygen uptake appeared to have a U-shaped association with birth weight (the smallest and largest children had the lowest uptake, *P* = 0.01), but this association was no longer significant after adjustment for parental BMI. Using the individual twin’s deviation from his own twin pair’s average birth weight, we found positive associations between birth weight and adolescent vertical jump (b = 3.49, *P* = 0.0007) and arm pull (b = 3.44, *P* = 0.02). Δ scores were calculated within the twin pairs as first born twin minus second born twin. Δ birth weight was associated with Δ vertical jump within MZ twin pairs only (b = 2.63, *P* = 0.009), which indicates importance of placental factors.

**Conclusions:**

We found evidence for an association between adolescent physical performance (strength, balance and possibly peak oxygen uptake) and birth weight. The associations with vertical jump and arm pull were likely based on individual, more specifically placental (in the case of vertical jump) factors. Our results should be viewed as hypothesis-generating and need confirmation, but potentially support preventive strategies to optimize birth weight, for example via placental function, to target later fitness and health.

## Introduction

Adult cardiorespiratory fitness is related to all-cause mortality and, more specifically, to cardiovascular mortality. [1], [2] Likewise, in adolescence and in young adults suboptimal cardiorespiratory fitness has been linked to unfavorable cardiovascular outcomes. [3]–[5] In addition, there is considerable interest in the developmental origins of cardiovascular fitness, which is mainly focused on the association with birth weight [6]–[8].

A number of studies suggest a positive association between birth weight and later cardiorespiratory fitness, which indicates that low birth weight babies may be programmed to have impaired cardiorespiratory fitness in adulthood. [6]–[8] In general, the fetus is thought to be plastic, adjusting itself to intrauterine circumstances in anticipation of its future, extrauterine environment. [9] A mismatch between intrauterine and extrauterine environment may give rise to disease. This may be the case when a growth-restricted fetus is born into the present Western society, where nutrients are abundantly available. Thus, the positive association between birth weight and adult cardiorespiratory fitness is in accordance with observations that low birth weight is related to cardiovascular disease and its risk factors later in life. [10]–[12] However, several studies failed to show an association between birth weight and cardiorespiratory fitness later in life. [13]–[15] These inconsistencies are possibly caused by methodological issues, such as the variety of measurement tools used to quantify cardiorespiratory fitness and by the different sets of potential confounders used in the analyses. Another possible explanation for these inconsistent findings is confounding by genetic factors. So far, the role of genetic factors in these associations has remained unclear.

To address the latter issue and to further explore the developmental origins of cardiorespiratory fitness, twin studies may be a useful tool, as they provide the possibility to unravel genetic and environmental (both fetoplacental and maternal) influences on the possible associations. We define maternal factors as all non-genetic maternal influences on both members of a twin pair, such as body mass index (BMI) and smoking. In dizygotic (DZ) twins, maternal factors are identical, but genetic factors are not, while in monozygotic (MZ) twins, both maternal and genetic factors are identical. In contrast to the maternal environment, the fetoplacental environment can be different for both members of a twin pair. A previous study used a twin sample to disentangle genetic and environmental factors in the association between birth weight and adult hand grip strength (a marker of muscle strength, which is also related to cardiovascular disease [16]) and found evidence for importance of genetic factors [17].

In the Leuven Longitudinal Twin Study (LLTS) we used a twin sample from the East Flanders Prospective Twin Survey (EFPTS), in which we measured cardiorespiratory fitness, as well as muscle strength, flexibility and balance, up to eight times during adolescence (10–18 years). We studied the associations between these variables and birth weight. Additionally, the importance of individual and shared factors was disentangled. Finally, we analyzed to what extent genetic, maternal or fetoplacental factors were involved.

## Subjects and Methods

### Participants

In a longitudinal study carried out from 1985–1999 (Leuven Longitudinal Twin Study) 114 twin pairs and their parents were recruited from the East Flanders Prospective Twin Survey (EFPTS), which is a population-based register of all twins born in the Belgian province of East Flanders since 1964. [18] All families with twins reaching the age of 10 years had been contacted by letter and had been further informed by telephone calls or home visits. The response rate was approximately 40%. The twins were first invited around the age of 10 years, and followed yearly to the age of 16 years, with one additional measurement at 18 years of age. At each visit anthropometric measurements were taken, motor performance was assessed by the Leuven Motor Test Battery and a maximum exercise test on a treadmill measured cardiorespiratory fitness (detailed description in the next section). The parents were assessed once, at the first visit.

### Ethics Statement

Both parents and twins gave written informed consent/assent. Ethical approval was given by the Medical Ethics Committee of the Fund for Medical Research and of the former Institute of Physical Education and Physiotherapy, KU Leuven.

### Variables

#### Physical fitness

Our primary outcome measure was peak oxygen uptake (VO_2_peak, measured in ml/min/kg) as obtained by a maximal exercise test on a treadmill (ELG2, Woodway, Waukesha, United States), according to the Bruce protocol. [19] Details of the test have been described previously. [20] Only results obtained at a heart rate >180 beats per minute were used in the analyses.

In addition, the test battery included measures of static strength (arm pull, in kilograms), explosive strength (vertical jump, in centimeters), muscle endurance/functional strength (bent arm hang, in seconds), muscle endurance/trunk strength (leg lifts, n in 20 seconds), running speed and agility (10 time 5 meters shuttle run test, in seconds), speed of limb movement (plate tapping test, n in 20 seconds), flexibility (sit-and-reach test, centimeter) and balance (flamingo balance test, n attempts to stand on one leg for one minute). Details of these tests have been described previously [21], [22].

#### Anthropometry

Body mass was measured to the nearest 0.1 kg using a balance scale (SECA 709, Hamburg, Germany) with subjects in underwear. Standing height was measured on bare feet using a Harpenden stadiometer (Holtain Instruments, Crymych, United Kingdom) to the nearest millimeter.

#### Perinatal characteristics

Birth weight, gestational age, sex, zygosity and chorionicity were collected prospectively. Zygosity was determined using sequential analysis based on sex, fetal membranes, blood groups, placental alkaline phosphatase and DNA marker analysis. Zygosity and chorionicity were analyzed as three groups: dizygotic (always dichorionic), monozygotic-dichorionic (MZDC) and monozygotic-monochorionic (MZMC) twins.

#### Potential confounders

As potential confounders we recorded smoking, physical activity and parental BMI. As a measure of physical activity, the average number of hours the children participated in sport activities expressed per week during the last year was recorded. At each visit smoking status was recorded and analyzed in three groups: current smoker, former smoker or never smoker. Parental weight and height were collected using the same methods as used in the twins during the first visit, and (parental) BMI was calculated as (weight in kilograms/height in meters^2^).

### Statistical Analysis

#### Descriptive analysis

In the descriptive analyses, for continuous variables differences between MZ and DZ twins were calculated by use of the t-test (normally distributed variables) or the Wilcoxon rank sum test (non-normally distributed variables), when appropriate. The *χ* ^2^ test was used to compare frequencies between MZ and DZ twins.

#### Twins as individuals

**Figure 1:**
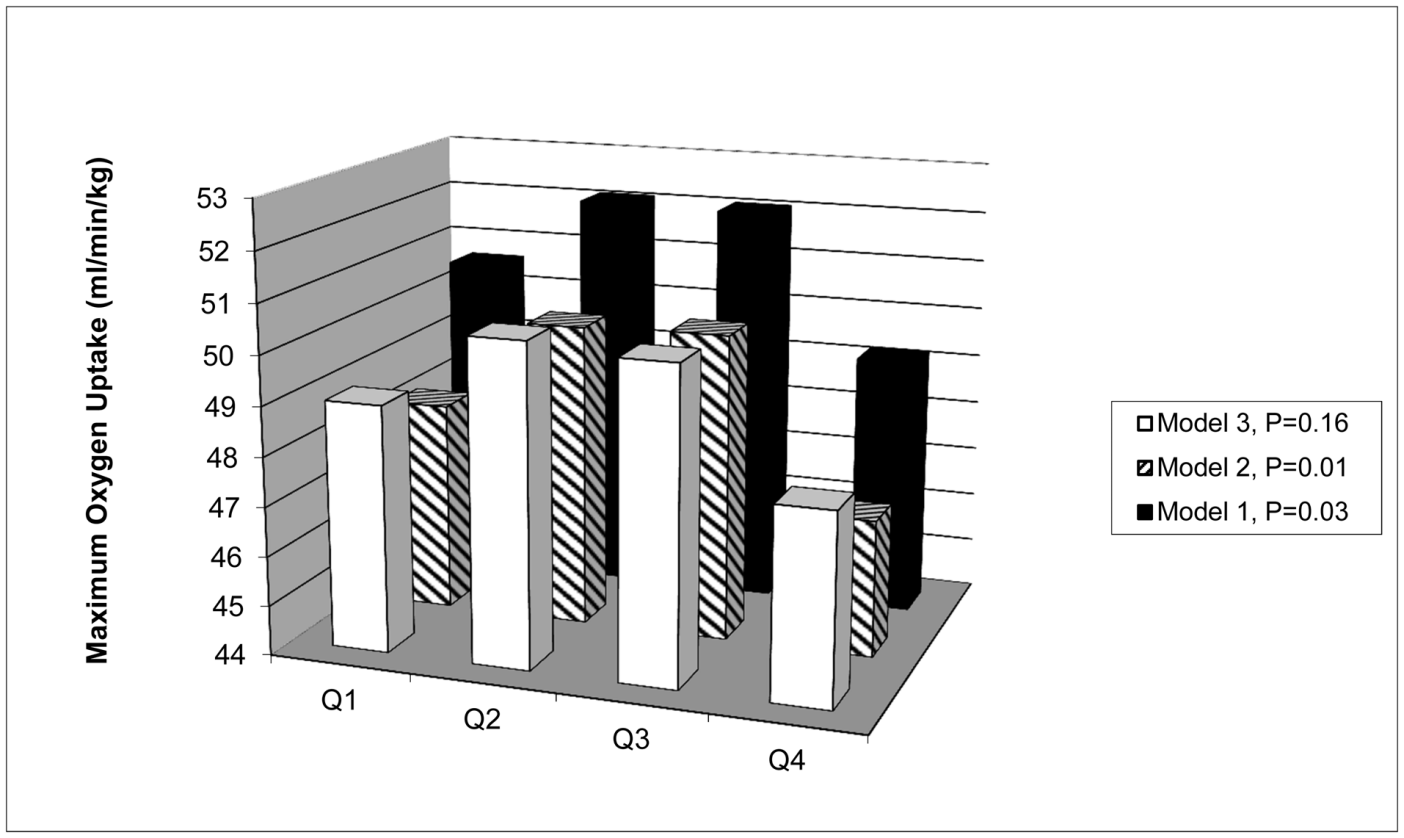
Average maximum oxygen uptake in birth weight quarters. Q1–Q4: Birth weight quarter 1–4 (Q1 = lowest quarter).

We used multivariable multilevel regression analysis to study the association between birth weight and the outcome variables. Failure to take the correlated nature of the data into account would lead to biased estimates and incorrect standard errors and *P*-values. Therefore, a three level random intercept model was used, taking into account the fact that the measurements were taken longitudinally in the same individuals (level 2), which were in turn clustered in twin pairs (level 3, highest level). The variance-covariance structure of DZ, MZDC and MZMC twins was allowed to differ. Three models were constructed: 1) basic model, adjusted only for sex, age (as time point) and gestational age, 2) as model 1, but adjusted also for child-specific factors: zygosity-chorionicity, smoking, physical activity, height and weight at the time of assessment, and 3) as model 2, but with additional inclusion of parental factors: maternal and paternal BMI. Since VO_2_peak was already calculated relative to weight, weight was not included in VO_2_peak models 2 and 3. The data used in figure 1 were calculated using the ‘least squared means’ function.

#### Individual versus shared factors

To evaluate the importance of individual (for example placental function) and shared factors (for example maternal characteristics) in the relationship between birth weight and the outcome parameters, separate within-twin-pair (using the individual twin’s birth weight deviation from the pair mean as a determinant) and between-pair regression coefficients (using the average birth weight of the pair as a determinant) were calculated.

#### Twins as pairs

To unravel genetic, maternal and placental influences on the associations, the same-sex twins were studied as pairs using a two level random intercept model, which takes the longitudinal measurements within the same individuals into account. We analyzed the association between intra-pair difference in birth weight and intra-pair difference in physical fitness for MZ and DZ twins separately. Since MZ twins share approximately 100% of their genes, genetic factors cannot be responsible for any differences observed within the pair and any association between birth weight and adult fitness observed within MZ pairs hence indicates environmental influences on the association. Since maternal factors are identical, but fetoplacental factors are never identical, these environmental influences can be pinpointed to fetoplacental factors. DZ twins share on average 50% of their genes and therefore intra-pair analysis in DZ twins is still partly confounded by genetic effects. The difference scores were calculated as first-born twin minus second-born twin (this yields the same results as subtracting the fastest from the slowest growing twin). [23] In the within-pair analyses, the difference in smoking status was not included in the model, because this categorical variable would yield too many possible difference scores for our moderate sample size. In the within-pair analyses, shared variables were not included in the models.

The analyses were performed in SAS 9.2 using SAS Enterprise Guide 4 (SAS institute, Cary, NC, USA). A *P*-value <0.05 was considered statistically significant.

## Results

A total of 114 twin pairs were analyzed **(** **Table 1** **)**. As expected, MZ twins were lighter than DZ twins at birth, but they were also of shorter gestational age. The MZ twins were slightly older and taller when the first measurement was performed and they smoked less at the last measurement. MZ twins performed slightly better on the sit-and-reach test and on the shuttle run at the age of 10.5 years. Approximately two thirds of the MZ twins were monochorionic (MC), while the percentage of boys and girls in the MZ and DZ groups was approximately equal.

**Table 1:** Twin Characteristics at the Start of the Study.

	MZ (n = 94)		DZ (n = 134)	
	N		n	
General Characteristics				
Age (Years)	92	10.5 [10.3, 10.6]	126	10.4 [10.1, 10.5]**
Birth Weight (Grams)	94	2401±509	134	2631±465**
Gestational Age (Weeks)	92	37.0 [34.0, 38.0]	124	38.0 [37.0, 39.0]**
Monochorionic (n, %)	94	66 (70.2)	134	0 (0)
Male (n, %)	94	46 (48.9)	134	66 (49.3)
Confounders				
Smoking-never (n, %)*	77	61 (79.2)	107	67 (62.6)**
Smoking-former (n, %)*	77	6 (7.8)	107	7 (6.5)
Smoking-current (n, %)*	77	10 (13.0)	107	33 (30.8)
Physical Activty (Hours per Week)	80	1.3 [0.0. 3.0]	105	1.0 [0.0. 2.0]
Height (m)	94	1.42 [1.35, 1.45]	134	1.39 [1.36, 1.43]**
Weight (kg)	94	32.0 [28.0, 34.4]	134	31.0 [28.1, 34.6]
Maternal BMI	94	22.8 [21.4, 25.7]	126	24.2 [22.3, 26.4]
Paternal BMI	88	25.0 [23.4, 27.3]	112	25.0 [23.1, 26.1]
Fitness Parameters				
V02 Peak (ml/min/kg, only Heart Rate >180 beats/min)	72	48.9±11.4	87	48.7±10.5
Vertical Jump (cm)	94	28.8±4.7	132	28.2±4.2
Arm Pull (kg)	94	25.7±4.3	132	24.7±4.7
Standing Long Jump (cm)	94	157 [145, 168]	132	155 [142, 165]
Situps (n in 20 s)	94	13 [10], [14]	132	13 [10], [14]
Flamingo Balance (n attempts to stand 1 minute)	94	16.6±5.5	132	17.1±6.5
Plate Tapping Test (n in 20 s)	94	63.9±6.2	132	62.0±8.2
Sit and Reach Test (cm)	94	22 [18], [26]	132	21 [15], [25] **
Lef Lifts (n in 20 s)	94	15 [13], [16]	132	15 [12], [16]
Bent Arm Hang (s)	92	10.5 [5.9, 16.0]	132	8.6 [4.2, 16.1]
Shuttle Run (s)	94	23.1 [22.0, 24.5]	132	22.5 [21.8, 24.0]**

* For description purposes, smoking was analyzed for the final measuring moment instead of at the start of the study.

Results are given as mean ± SD, median [interquartile range] or as n (%).

MZ: monozygotic, DZ: dizygotic,

** Comparison MZ and DZ twins <0.05 (t-test).

### Twins as Individuals

**Table 2** shows the regression coefficients obtained from our three different models in which the twins were regarded as individuals. Peak oxygen uptake showed a negative association with birth weight in models 1 and 2: b = −2.53 ml/min/kg per kg birth weight (*P* = 0.04) and b = −2.74 (*P* = 0.04), respectively. Peak oxygen uptake was not related to birth weight in the fully adjusted model (model 3). To further explore the unanticipated negative associations in models 1 and 2, we extended the analysis to study birth weight quartiles. The highest birth weight quarter was used as the reference category. We identified significant differences between the birth weight quartiles in models 1 and 2, and a similar, though non-significant, data structure in model 3 **(** **Figure 1** **)**. The data in figure 1 represent the expected peak oxygen uptake for a hypothetical average participant of the study in a specific quartile. The corresponding values for model 2 were: smallest quarter 48.2 ml/min/kg (*P* = 0.36 from largest quarter), second quarter 50.1 ml/min/kg (*P* = 0.01), third quarter 50.2 ml/min/kg (*P* = 0.004) and the largest quarter: 46.8 ml/min/kg, overall *P* = 0.01. For model 3, the corresponding values were: smallest quarter 49.0 ml/min/kg (*P* = 0.52 from largest quarter), second quarter 50.5 ml/min/kg (*P* = 0.06), third quarter 50.3 ml/min/kg (*P* = 0.07) and the largest quarter: 47.8 ml/min/kg, overall *P* = 0.16. There were no other non-linear associations identified in models 2 or 3.

**Table 2:** Regression Coefficients for Birth Weight in the Individual Analysis.

	Birth Weight (kg)									
	Model 1			Model 2			Model 3			
	n	b	*P*	n	b	*P*	n	b	*P*	
V02 Peak (ml/min/kg)	108	−2.53	0.04	100	−2.74	0.04	85	−2.01	0.16	
Vertical Jump (cm)	108	1.25	0.11	100	1.38	0.09	85	1.96	0.02	
Arm Pull (kg)	108	3.44	0.0002	100	1.35	0.10	85	1.85	0.03	
Standing Long Jump (cm)	108	−0.26	0.92	100	−1.93	0.47	85	−0.76	0.79	
Situps (n in 20 s)	108	0.25	0.64	100	0.60	0.28	85	0.76	0.23	
Flamingo Balance (n attempts to stand 1 minute)	108	−0.71	0.35	100	−1.36	0.08	85	−1.82	0.03	
Plate Tapping Test (n in 20 s)	108	1.55	0.16	100	0.76	0.50	85	1.59	0.18	
Sit and Reach Test (cm)	108	0.17	0.87	100	−0.96	0.37	85	−0.59	0.59	
Lef Lifts (n in 20 s)	108	−0.74	0.04	100	−0.60	0.10	85	−0.24	0.55	
Bent Arm Hang (s)	108	−0.21	0.91	100	1.83	0.38	85	4.39	0.052	
Shuttle Run (s)	108	0.12	0.54	100	0.03	0.89	85	−0.17	0.45	

Results were obtained by use of a three-level random intercept, random slope (for time) model.

Model 1: birth weight, gestational age, time point and sex.

Model 2: model 1+zygosity-chorionicity, smoking, physical activity, height and weight (the latter not included in the V02 peak models).

Model 3: model 2+parental BMI.

In the fully adjusted models using birth weight as a continuous variable adolescent vertical jump (b = 1.96 cm per kg birth weight, *P* = 0.02), arm pull (b = 1.85 *P* = 0.03) and flamingo balance (b = −1.82, *P* = 0.03) were related to birth weight. In all three cases, higher birth weight was associated with better performance, since performance on the flamingo balance is better when fewer attempts are needed to complete the test **(** **Table 2** **)**.

When the twin pair’s average birth weight was used as the independent variable in the fully adjusted model, no associations were identified **(** **Table 3** **).** On the other hand, using the individual twin’s deviation from his own twin pair’s average birth weight, we found positive associations between birth weight and adolescent vertical jump (b = 3.49, *P* = 0.0007) and between birth weight and adolescent arm pull (b = 3.44, *P* = 0.02).

**Table 3:** Regression Coefficients for Birth Weight: Mean of the Twin Pair and Difference within the Twin Pair.

		Birth Weight Mean (kg)		Birth Weight Difference (kg)	
	n	b	*P*	b	*P*
V02 Peak (ml/min/kg)	85	−2.02	0.40	−2.01	0.25
Vertical Jump (cm)	85	−1.30	0.36	3.49	0.0007
Arm Pull (kg)	85	0.96	0.35	3.44	0.02
Standing Long Jump (cm)	85	−6.59	0.16	2.51	0.49
Situps (n in 20 s)	85	−0.04	0.97	1.15	0.15
Flamingo Balance (n attempts to stand 1 minute)	85	−1.85	0.11	−1.80	0.13
Plate Tapping Test (n in 20 s)	85	0.79	0.64	2.29	0.16
Sit and Reach Test (cm)	85	0.04	0.98	−0.90	0.51
Lef Lifts (n in 20 s)	85	0.48	0.46	−0.68	0.18
Bent Arm Hang (s)	85	5.31	0.16	3.89	0.16
Shuttle Run (s)	85	0.49	0.19	−0.51	0.06

Results were obtained by use of a three-level random intercept, random slope (for time) model.

Model: birth weight, gestational age, time point, sex, zygosity-chorionicity, smoking, physical activity, height, weight (not included in the V02 peak models) and parental BMI.

### Twins as Pairs

When MZ and DZ twins were analyzed separately, no associations were found within DZ twins. Several associations were observed in MZ twins in the basic model: birth weight was positively related to vertical jump, arm pull, standing long jump, and plate tapping, while it was inversely related to flamingo balance and shuttle run **(** **Table 4** **)**. One association remained significant in the fully adjusted model: Δ birth weight was associated with Δ vertical jump, in MZ twins only (b = 2.63, *P* = 0.009).

**Table 4:** Regression Coefficients for Δ Birth Weight in MZ and DZ twins (Pair Wise, Stratified Analysis).

	Δ Birth Weight (kg)							
	Model 1				Model 2			
	MZ (n = 47)		DZ (N = 43)		MZ (N = 44)		DZ (N = 41)	
	B	*P*	B	*P*	B	*P*	B	*P*
Δ V02 Peak (ml/min/kg)	−1.38	0.38	−1.78	0.48	−1.06	0.63	−0.04	0.99
Δ Vertical Jump (cm)	3.31	0.0002	1.66	0.28	2.63	0.009	1.12	0.50
Δ Arm Pull (kg)	2.92	0.04	4.15	0.06	1.14	0.45	0.11	0.95
Δ Standing Long Jump (cm)	8.11	0.04	−2.56	0.61	−1.26	0.78	−5.68	0.32
Δ Situps (n in 20 s)	−0.12	0.86	1.36	0.15	−0.30	0.73	1.28	0.20
Δ Flamingo Balance (n attempts to stand 1 minute)	−3.95	0.004	−1.36	0.44	−2.10	0.17	−2.75	0.15
Δ Plate Tapping Test (n in 20 s)	4.06	0.009	−0.58	0.84	0.42	0.80	0.35	0.89
Δ Sit and Reach Test (cm)	2.10	0.13	−0.51	0.83	1.07	0.49	−0.84	0.74
Δ Lef Lifts (n in 20 s)	−0.37	0.51	−0.38	0.60	−0.19	0.75	−0.20	0.79
Δ Bent Arm Hang (s)	−1.86	0.43	−1.61	0.56	−2.33	0.47	0.71	0.80
Δ Shuttle Run (s)	−0.67	0.03	−0.15	0.74	−0.37	0.29	−0.19	0.69

Results were obtained by use of a two-level random intercept, random slope (for time) model.

Model 1: Δ birth weight and time point.

Model 2: model 1+chorionicity, Δ physical activity, Δ height and Δ weight (the latter not included in the V02 peak models).

MZ: Monozygotic, DZ: Dizygotic.

## Discussion

In the present prospective, longitudinal, twin study we found that the lowest and highest birth weight groups had the lowest peak oxygen uptake in adolescence (i.e. a U-shaped association). However, after adjustment for parental BMI the effect size was attenuated and the association was no longer significant. We hypothesized a lesser performance by children small at birth, but apparently also high birth weight children are at risk. It is not clear what the exact mechanistic meaning of adjustment for parental BMI is. We speculate that parental BMI may be a surrogate for a sedentary lifestyle, poor nutritional habits, genetic predisposition for obesity, low educational level, or a combination of the above. It is most likely also related to birth weight, given the confounding characteristics. Given the similar data structure in models 2 and 3 and the accompanying loss of 15 twin pairs, model 3 was potentially influenced by a statistical power problem.

From the other variables studied not only static (arm pull) and explosive (vertical jump) strength were associated with birth weight, but also a more complex measure as balance appeared to be associated with birth weight. In all cases, low birth weight was associated with a lesser performance in adolescence, thereby supporting developmental programming by low birth weight. Importantly, muscle strength, at least as measured by grip strength, is inversely related to cardiovascular and all-cause mortality. [24], [25] With regard to the strength variables our results are in accordance with the literature. [26], [27] Furthermore, there is prior evidence that balance is positively related to birth weight in elderly men [28].

Furthermore, our study shows that the association between birth weight and static and explosive strength is most likely due to individual (genetic or fetoplacental) factors, as the association was observed using the twin’s individual deviation of the pair’s mean birth weight. This means that having a lower birth weight than one’s co-twin results in lower adult strength than one’s co-twin and hence this cannot be the result of a shared factor. This result is contrary to the only other study in this field, which concluded that genetic factors were most likely to be important in the association between birth weight and, in this case, adult hand grip strength. [17] In this study, however, a different outcome variable was studied at a different age (25 years). In the case of vertical jump, we were more specifically able to show a likely importance of fetoplacental factors, as can be inferred from the fact that we observed the association within MZ twin pairs, who have identical genetic and maternal characteristics. Hence, genetic and maternal factors cannot cause the association and fetoplacental factors are the only remaining explanation. Placental factors are known to play a role in the developmental origins of health and disease as the placenta is the primary organ to regulate oxygen and nutrients to the developing fetus. [29] This means that fetal adaptations (plasticity) are partly made based on placental function. For example, placental inefficiency was shown to relate to childhood blood pressure. [30] Altogether, our data are supportive of preventive strategies during pregnancy, such as prevention of maternal smoking and promotion of healthy maternal diet and body composition to optimize placental function. In addition, our data are supportive of ‘environmental’ hypotheses regarding developmental programming, such as the thrifty phenotype hypothesis, and do not support the fetal insulin hypothesis, which assumes a common genotype for both birth weight and adult cardiovascular disease [31], [32].

As a limitation, extrapolation of birth weight studies from twins to singletons should be done with caution. Twins have lower birth weights and shorter gestational ages than singletons, and their prenatal environment is different from that of singletons. [33] Second, our study represents a simplification of reality, because we treat MZ twins as 100% genetically identical in our analyses. We know that post-zygotic copy number variations (CNV’s) and single nucleotide polymorphisms (SNP’s) are highly concordant in MZ twins. [34] However, we did not take epigenetic drift, which constitutes that MZ twins have increasing intra-pair differences in epigenetic profiles with age, into account. These differences are detectable at birth, and increase with age, although their crude magnitude is small and highly variable, depending on the genetic region. [34]–[37] It seems reasonable to expect a small amount of epigenetic differences within our young MZ twins, which we did not measure. Third, we present a study of a moderate sample size, which increased the risk for a type 1 error. Although the outcome variables were selected by factor analysis and therefore represent different entities within the concept of physical fitness, the risk for a type 1 error is still present. [38] Since our results would not withstand multiple testing adjustment, they should be viewed as hypothesis-generating and therefore need confirmation in other studies.

As important advantages, we present a study in which we repeatedly measured the same broad spectrum of outcome parameters in the same individuals. This yields increased precision in the measurements and provides a rather complete overview of different fitness, strength and performance parameters. Birth weight was thereby collected prospectively.

In summary, we found evidence for an association between adolescent physical performance (strength, balance and possibly peak oxygen uptake) and birth weight. The associations with vertical jump and arm pull were most likely based on individual, more specifically placental (in the case of vertical jump), factors. Our results should be viewed as hypothesis-generating, but potentially support early preventive strategies to optimize birth weight to target later physical performance and probably cardiovascular health. This might be possible by prevention of smoking and by promotion of healthy diets for pregnant women to improve placental function. These possible developmental strategies should be applied together with attempts to improve cardiorespiratory fitness through life style changes in adult patients at risk. With regard to cardiorespiratory fitness, more research is needed, especially on the exact meaning of adjusting these types of data for parental BMI.
